# Pathological Features and Clinical Characteristics of Kikuchi-Fujimoto Disease: A Tertiary Hospital Experience in Riyadh, Saudi Arabia

**DOI:** 10.7759/cureus.33683

**Published:** 2023-01-12

**Authors:** Saeed AlShieban, Emad Masuadi, Rayan Alghamdi, Abdulrahman Alshalfan, Saud Alessa, Almohannad K Alqarni, Zeyad Alotaibe, Hanaa Bamefleh

**Affiliations:** 1 Pathology, King Abdullah International Medical Research Center, Riyadh, SAU; 2 Pathology, Ministry of National Guard Health Affairs, Riyadh, SAU; 3 Pathology, King Saud Bin Abdulaziz University for Health Sciences, Riyadh, SAU; 4 Research Unit/Biostatistics, King Saud Bin Abdulaziz University for Health Sciences, Riyadh, SAU; 5 Research Unit/Biostatistics, King Abdullah International Medical Research Center, Riyadh, SAU; 6 College of Medicine, King Saud Bin Abdulaziz University for Health Sciences, Riyadh, SAU

**Keywords:** lymphadenopathy, necrotizing histiocytic lymphadenitis, lymphoma, lymph node biopsy, kikuchi–fujimoto

## Abstract

Background

Kikuchi-Fujimoto disease (KFD) - also known as necrotizing histiocytic lymphadenitis - is a benign histiocytic lymphadenitis known for its low incidence and misdiagnosis that occurs mostly in young Asian females more than males. This disease resolves spontaneously in a few months with a low risk of relapse (one in 30 patients) after resolution.

Objectives

The aim of this study is to share King Abdulaziz Medical City's (KAMC's) experience with KFD by determining its clinicopathological characteristics.

Materials and methods

In this study, we reviewed histopathological slides and pathological reports of all lymphadenopathy cases (683 cases) in the period between January 2008 and December 2018.

Results

Forty-four cases of KFD were found and their clinicopathological characteristics were recorded. There is a slight female predominance (59% females versus 41% males) with a wide age range from 10 months to 97 years (mean = 28.8). The majority of the cases (63.6%) are seen in young adults (between 21 and 40 years). Association with autoimmune diseases was shown by 20.5% of cases while viral infection association was shown by few cases. Most cases showed remission (59%) and no deaths were reported upon follow-up. Histopathologically, the majority of cases have proliferative type followed by the necrotic type and few cases showed xanthomatous type.

Conclusions

Our study has the largest number of KFD cases in this region. It is obvious that KFD has clinical, radiological, and pathological features that overlap with malignancy, especially lymphoma. Knowing this disease and careful diagnostic approach can help avoid misdiagnosis.

## Introduction

Kikuchi-Fujimoto disease (KFD) - also known as necrotizing histiocytic lymphadenitis - is a benign histiocytic/dendritic proliferation known for its low incidence and misdiagnosis that occurs predominantly in young females [1-3]. This disease resolves spontaneously in a few months with a low risk of relapse (one in 30 patients) after resolution [4]. The geographic territories may correspond to human leukocyte antigen (HLA) alleles such as HLA class 2 alleles (HLA-DPB1 and HLA-DPBA) which were more dominant in Asian patients with KFD while being extremely rare or even absent among Caucasian patients [1]. KFD has three types, proliferative type, necrotic type, and xanthomatous (foamy cell) type [5]. The proliferative type is the most common type and is characterized by the proliferation of a large number of medium-to-large lymphoid cells, histiocytes, and dendritic cells with variable apoptosis and activation of phagocytosis. The necrotic type is characterized by wide areas of necrosis surrounded by the cellular component. The xanthomatous type is characterized by aggregates of foamy histiocytes surrounding the necrosis. Both necrotic and xanthomatous types are less common compared to the proliferative ones. Although most cases resolve spontaneously, some cases showed resistance even after treatment with steroids and immunosuppressive agents [6].

It is obvious that KFD has clinical, radiological, and pathological features that overlap with malignancy, especially lymphoma. Knowing this disease and careful diagnostic approach can help avoid misdiagnosis [7].

The aim of this study is to share King Abdulaziz Medical City's (KAMC's) experience with KFD by determining its clinicopathological characteristics.

## Materials and methods

Patients

This was a retrospective observational study conducted at the Department of Pathology and Laboratory Medicine, King Abdulaziz Medical City, National Guard Health Affairs, Riyadh, Saudi Arabia. We reviewed histopathological slides and pathological reports of all lymphadenopathy cases (683 cases) in the period between January 2008 and December 2018.

Clinical review

Clinical findings and serological test results were reviewed to exclude systemic lupus erythematosus (SLE) which sometimes has similar histopathological findings as KFD. Clinical and demographic data of KFD patients were reviewed, including age, gender, lymph node location, lymph node size, and presence or absence of constitutional symptoms.

Tissue preparation and histopathological review

The archived histopathological samples were reviewed. They include tissue biopsies from suspicious lymph nodes. All biopsies were fixed in 10% buffer formalin for an average of 16 hours, then grossed by pathology residents or authorized histotechnologists, then processed overnight using Sakura Tissue-Tek VIP® processor. The formalin-fixed, paraffin-embedded tissue was used to prepare hematoxylin and eosin (H&E)-stained slides and unstained slides to be ready for immunohistochemical stains when needed. Initially, all H&E-stained slides were reviewed for the characteristic features of KFD. These features include the proliferation of a large number of medium-to-large lymphoid cells, histiocytes, and dendritic cells with variable apoptosis and activation of phagocytosis, and wide areas of necrosis surrounded by aggregates of foamy macrophages.

Immunohistochemical study

The immunohistochemical staining was performed using the avidin-biotin-peroxidase complex method on Ventana BenchMark Ultra (Roche Diagnostics, Basel, Switzerland). The antibodies used are a cluster of differentiation (CD)20 (clone L26), PAX-5 (clone SP34), CD3 (clone MRQ-39), CD5 (clone 4C7), CD4 (clone SP35), CD8 (clone C8/144B), CD30 (clone Ber-H2), CD15 (clone MMA), myeloperoxidase (polyclonal), CD123 (clone IL3RA) , CD68 (clone KP1), Ki-67 (clone 30-9), cytomegalovirus (CMV) (clone CCH2+DDG9), and human herpesvirus 8 (HHV-8) (clone 13B10), and in situ hybridization for Epstein-Barr virus (EBV). The immunostaining result is further categorized into negative (score 0), weak (score 1), moderate (score 2), and strong (score 3).

All histopathological materials were reviewed by the principal investigator who is a certified hematopathologist to ensure the accuracy of the diagnosis.

Statistical analysis

As this is a descriptive study, data were presented as mean for continuous variables, such as the size of lymph nodes, and frequencies (percentages) for categorical variables, such as gender.

## Results

During the study period, a total of 44 cases were diagnosed as KFD out of 683 lymph node biopsies done during the search period with an incidence of 6.4%.

Table 1 summarizes the clinical characteristics of KFD cases. There is a slight female predominance (59% females versus 41% males). KFD is seen in a wide age range from 10 months to 97 years (mean = 28.8). The majority of the cases (63.6%) are seen in young adults (between 21 and 40 years). Most of the involved lymph nodes are cervical (70.5%) followed by axillary (16%) and inguinal (9%) lymph nodes. The site of involved lymph nodes was not indicated in two cases. No extranodal involvement was identified. There is a wide range of the size of involved lymph nodes between 0.9 and 14 cm (mean = 3.5 cm). Investigation for the associated clinical conditions, mainly viral infection, autoimmune diseases, and malignancy, showed that EBV was detected in five cases (11.4%), CMV in four cases (9.1%), and Erythroparvovirus in one case (2.3%). Autoimmune diseases (three Hashimoto’s thyroiditis, three type 1 diabetes mellitus, one Graves disease, one rheumatoid arthritis, and one Sjögren’s syndrome) were seen in nine cases (20.5%) after excluding SLE. No malignancy was detected in KFD cases. Upon follow-up, 26 patients had either complete or partial remission (59%). Six patients (14%) developed other clinical conditions (diabetes mellitus type 2 [two patients], hypertension [two patients], nephrotic syndrome [one patient], and gallstones [one patient]). Three patients (7%) died from other causes (myocardial infarction [two patients] and septicemia [one patient]). No follow-up was available for nine patients (20%).

**Table 1:** Clinical characteristics of Kikuchi-Fujimoto disease cases EBV, Epstein-Barr virus; CMV, cytomegalovirus.

Clinical Characteristics	Findings	Number (n)	Percentage
Gender	Female	26	59
	Male	18	41
Age	Range (10 months-97 years)	-	-
	Mean (28.8 years)	-	-
	Peak (21-40 years)	28	63.6
Lymph node size	0.9-14 cm (mean = 3.5 cm)	-	-
Lymph node site	Cervical	31	70.5
	Axillary	7	16
	Inguinal	4	9
	Unknown	2	4.5
Associated conditions	EBV	5	11.4
	CMV	4	9.1
	Erythroparvovirus	1	2.3
	Autoimmune disease	9	20.5
Outcome	Remission	26	59
	Developed other conditions	6	14
	Died from other diseases	3	7
	No follow-up	9	20

Fever, cachexia, upper respiratory tract (URT) symptoms, and splenomegaly are frequent clinical presentations/findings at the time of diagnosis (48%, 34%, 30%, and 25%, respectively). Other symptoms like lethargy, nausea, vomiting, hepatomegaly, arthralgia, pallor, and diarrhea are less frequent presentations.

Table 2 summarizes the histopathological types of KFD. The majority of cases are proliferative type (54.5%), followed by necrotic type (31.8%), and few cases show xanthomatous type (13.7%).

**Table 2:** Histopathological types of Kikuchi-Fujimoto disease cases

Type	Number	Percentage
Type 1: Proliferative type	24	54.5
Type 2: Necrotic type	14	31.8
Type 3: Xanthomatous type	6	13.7
Total	44	100

**Figure 1:**
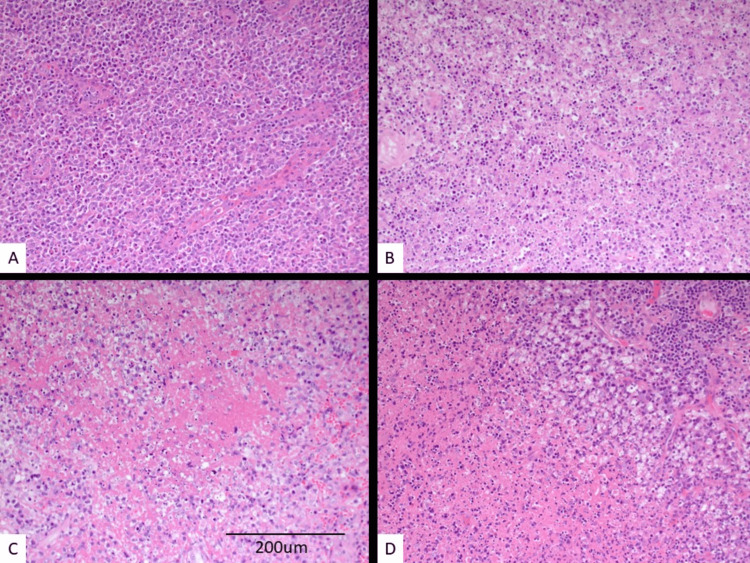
Morphological patterns of Kikuchi-Fujimoto disease Different morphological features according to the KFD type. Proliferation of large lymphoid cells, histiocytes, and dendritic cells (A, 200×, H&E stain), with variable apoptosis and activation of phagocytosis (B, 200×, H&E stain) in type 1. Wide areas of necrosis in type 2 (C, 200×, H&E stain). Foamy histiocytes in type 3 (D, 200×, H&E stain). KFD, Kikuchi-Fujimoto disease; H&E, hematoxylin and eosin.

Histopathological features of KFD are shown in Figure 1. In type 1, there is the proliferation of a large number of medium-to-large lymphoid cells, histiocytes, and dendritic cells with variable apoptosis and activation of phagocytosis (Figure 1A, 1B). The necrotic type is characterized by wide areas of necrosis surrounded by the cellular component (Figure 1C) while the xanthomatous type is characterized by aggregates of foamy histiocytes surrounding the necrosis (Figure 1D).

**Figure 2:**
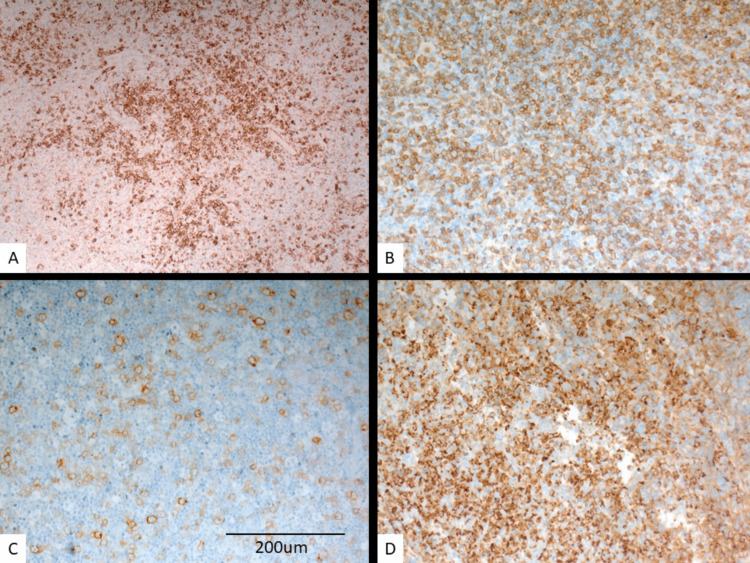
Immunophenotypical features of Kikuchi-Fujimoto disease Strong (3+) CD123 expression by plasmacytoid dendritic cells (A, 200×). Strong (3+) CD3 expression by lymphoid cells and immunoblasts (B, 200×) and CD30, moderate, 2+ (C, 200×). Strong (3+) myeloperoxidase expression by histiocytes (D, 200×).

Figure 2 shows the classical immunophenotype of the different cellular components of the disease. The plasmacytoid dendritic cells are characterized by strong (3+) CD123 immunostain expression (Figure 2A). The large lymphoid cells and immunoblasts are predominantly T cells expressing CD3, strong (3+) (Figure 2B) and CD30, moderate (2+) (Figure 2C) immunostains while the histiocytes in this disease are interestingly expressing myeloperoxidase immunostain (strong, 3+) (Figure 2D).

## Discussion

KFD is a self-limiting benign disorder seen more commonly in Asian patients, which, however, can be seen in a wide geographic distribution [1-3]. 

In Saudi Arabia, around 11 studies and case reports were conducted exploring the clinicopathological characteristics of KFD. Two studies (Abba A et al. in 1995 and Al-Maghrabi J et al. in 2005) were done on a large scale [7,8]. They showed an incidence of 0.6% and 1.16% KFD out of 2500 and 1884 lymphoid tissue biopsies, respectively. Since this illness is rare and the etiology behind it is not well comprehended, many case reports suggested a relation between this disease and developing an autoimmune disease such as Sjogren’s syndrome, Hashimoto’s thyroiditis, and pseudoappendicitis [9-13]. In addition, there are some hypothesized etiologies including viral infections such as EBV, parvovirus B-19, HHV8, human herpesvirus 6, and CMV [11-14]. Moreover, KFD has been linked with antiphospholipid syndrome, polymyositis, bilateral uveitis, peripheral arthritis, cutaneous necrotizing vasculitis, and pulmonary hemorrhage [15]. A familial predisposition has been reported, and one case developed KFD after splenectomy surgery [16,17].

According to the literature, this disease shows positive autoimmune tests, with elevated D-dimer, activated partial thromboplastin clotting time (aPTT), prothrombin time (PT), international normalized ratio (INR), C-reactive protein (CRP), erythrocyte sedimentation rate (ESR), lactate dehydrogenase (LDH) levels, and inflammatory markers and with low fibrinogen, hemoglobin, and blood cell counts, while peripheral blood smear shows evidence of schistocytes [3,14,18,19]. On the other hand, patients can present with extranodal or nodal involvement. The affected lymph nodes are typically tender, soft to firm in nature, with cortical and paracortical involvement [2,5,17]. Typically, patients present with unilateral lymphadenopathy and mostly involve cervical lymph nodes in 70%-98% of cases followed by axillary (14%) and supraclavicular (12%) lymph nodes [20]. Furthermore, according to a study conducted in Israel, retroperitoneal lymph node involvement is more common than cervical lymph node in their analysis suggesting a geographic/genetic difference [21]. Other unique presentations of KFD include splenomegaly, hepatomegaly, upper respiratory complaints, lethargy, gradual onset of pallor, arthralgia, cachexia, nausea, vomiting, diarrhea, and persistent fever [5,14,22-24]. It is also noted that KFD has similarity and confusion with some autoimmune diseases such as SLE and some infectious diseases such as toxoplasma lymphadenitis, cat scratch disease, and tuberculosis [5,25].

Radiological investigations of KFD by positron emission tomography/computed tomography (PET/CT) showed increased metabolic activity similar to that seen in malignant diseases such as lymphoma [26,27]. The necessity for lymph node biopsy increases as misdiagnosis is being observed in up to 40% of cases by the majority of healthcare professionals on a wide range [27,28]. The pathological evaluation shows variable histologic features corresponding to the type of the disease. Generally, there are patchy areas of non-granulomatous necrosis with marked apoptosis and nuclear debris (karyorrhexis) associated with aggregates of histiocytes, activated T immunoblasts, and plasmacytoid dendritic cells. Classically neutrophils and eosinophils are absent. CD8-positive T-lymphocytes are predominant [5,18,19].

In our study, we reported 44 cases of KFD after excluding SLE cases as they show almost similar histopathological findings; however, they have distinctive clinical course, management, and outcome. We found a 6.4% incidence compared to 0.6% and 1.16% reported in Abba A et al. in 1995 and Al-Maghrabi J et al. in 2005 [7,9]. The increased risk may be related to the awareness of healthcare providers about the disease and the availability of diagnostic tools like immunohistochemistry. Another possible reason is the association with infectious causes and autoimmune diseases. Previous studies showed that young adult females are mostly affected by the disease [1-3]. Similarly, this is observed in our study. There is female predominance seen in our patients (59% versus 41%). The disease is affecting patients in a wide age range. The youngest patient in our study is a 10-month-old boy and the oldest is a 97-year-old man; however, the mean age is around 28.8 years. In addition, when we distribute our patients in intervals of 20 years each, the majority are young adults between 21 and 40 years old (63.6%). Most of our patients present with fever, cachexia, URT symptoms, and splenomegaly similar to those observed before [5,14,22-24]. However, lethargy, nausea, vomiting, hepatomegaly, arthralgia, pallor, and diarrhea are less frequently noticed. Cervical lymph nodes remain the most common site of involvement followed by axillary lymph nodes [2]. Furthermore, four of our cases show inguinal lymph node involvement which is an unusual location that may be related to geographic or genetic differences. The finding of an unusual site of involvement is previously observed where retroperitoneal lymph node involvement was reported as the most commonly affected site in the study done in Israel [21]. We did not see an extranodal site of involvement in our study. There is also a wide range of lymph node sizes at the time of diagnosis. The smallest lymph node identified was 0.9 cm while the largest one was 14 cm (the mean size was 3.5 cm), which can be confusing with malignancy.

It is known that KFD can be associated with other medical illnesses - like viral infection, autoimmune diseases, and malignancy - or idiopathic [9-17]. Most of our cases are idiopathic (56.7%) and 43.3% show association with predominantly autoimmune diseases and less commonly viral infection. EBV and CMV are the most frequent viruses detected and only one case showed an association with Erythroparvovirus.

As expected, our cases showed benign clinical course during follow-up. The majority have complete or partial remission without major medical complications or deterioration. No disease-related deaths were detected.

Histopathologically, all three morphological types were observed in our cases [5]. Type 1 (proliferative) remains the most common type followed by the necrotic type while the xanthomatous type is less frequently seen. The variable morphological pattern especially with the presence of large lymphoid cells, immunoblasts, plasmacytoid dendritic cells, apoptosis, and necrosis increases the risk of misdiagnosis as lymphoma [27-30]. Careful morphological evaluation with the use of appropriate immunohistochemical stains can help in avoiding this problem.

There are some limitations to our study, such as the small sample number and being a single-center-based study which may not reflect the entire region.

## Conclusions

This study has the largest number of cases from Saudi Arabia. Although KFD is a benign condition, it carries clinical and pathological features that can overlap with other serious medical conditions like malignancy. Awareness of healthcare providers (clinicians and pathologists) about the disease can help avoid this dilemma.
